# Update on Genetic Conditions Affecting the Skin and the Kidneys

**DOI:** 10.3389/fped.2018.00043

**Published:** 2018-03-02

**Authors:** Antonia Reimer, Yinghong He, Cristina Has

**Affiliations:** ^1^Department of Dermatology, Faculty of Medicine, Medical Center, University of Freiburg, Freiburg, Germany; ^2^Berta-Ottenstein-Programme, Faculty of Medicine, University of Freiburg, Freiburg, Germany

**Keywords:** epidermolysis bullosa, mosaicism, genodermatosis, kidney, mutation, RASopathy, nevus, renal anomaly

## Abstract

Genetic conditions affecting the skin and kidney are clinically and genetically heterogeneous, and target molecular components present in both organs. The molecular pathology involves defects of cell–matrix adhesion, metabolic or signaling pathways, as well as tumor suppressor genes. This article gives a clinically oriented overview of this group of disorders, highlighting entities which have been recently described, as well as the progress made in understanding well-known entities. The genetic bases as well as molecular cell biological mechanisms are described, with therapeutic applications.

## Introduction

Anomalies of both skin and kidney occur in a vast number of genetic conditions. There are two major reasons for this concomitant occurrence of clinical manifestations. First, skin and kidney share a common embryological background represented by mesoderm for dermal connective tissue and kidneys. Second, various molecules involved in adhesion (e.g., integrin α3, CD151), cholesterol biosynthesis (e.g., NSDHL), or signaling pathways [e.g., Wnt, hedgehog (Hh), Ras/MAPK] are highly relevant for the development, structure, and function of both organs. In some syndromes, cutaneous and renal involvements are among the striking, pathognomonic features. Many of these disorders are recognizable at birth or early childhood.

Renal anomalies include congenital abnormalities of the kidney and urinary tract (CAKUT) (e.g., renal hypoplasia or aplasia, horseshoe deformations, anomalies of the urine collection system), malfunctioning of glomerular filtration, and the predisposition for tumors. The spectrum of skin anomalies is wide including pigmentation anomalies, skin dryness and ichthyosis, vascular anomalies (e.g., nevi flammei and hemangiomas), benign and malign skin tumors, abnormal hair, and nail dystrophy.

In this overview, genetic conditions affecting the skin and the kidneys are divided into three main groups:

1. Monogenic disorders with skin and renal involvement
2. Disorders due to postzygotic mosaicism
3. Chromosomal aberrations.

## Monogenic Disorders with Skin and Renal Involvement

In certain monogenic disorders, such as epidermolysis bullosa (EB), RASopathies or disorders with tumor predisposition, cutaneous, as well as primary or secondary renal involvement may occur. In this section, we update the clinical and molecular features of the most relevant disorders of this vast group. In a large number of other genodermatoses and genetic syndromes, renal involvement may occur, but is not a defining feature. The clinical and molecular characteristics of these rare disorders are updated in Tables 1 and 2.

**Table 1:** Genodermatoses with reno-urinary involvement.

Disorder MIM	Kidney involvement and its frequency(% of cases), if known	Skin involvement and its frequency(% of cases), if known	Affected gene and protein	Incidence	Onset of clinical manifestations	Inheritance
Restrictive dermopathy 275210	Urethral duplication occasional	Skin is thin, translucent and forms a tight, rigid casing. Erosions and fissures occur mainly in folds 100	*ZMPSTE24* and *LAMNA* mutations, leading to abnormal function of lamin A	Approximately 60 cases reported	Prenatal/at birth	AR (*ZMPSTE24*) less frequently *de novo* dominant (*LMNA*)

Nephrosis with ichthyosis and adrenal insufficiency	Steroid-resistant nephrotic syndrome major feature	IchthyosisMajor feature	*SGPL1*, sphingosine-1-phosphate lyase	NA	Early adulthood	AR

Arthrogryposis-renal dysfunction-cholestasis (ARC) syndrome 208085	Renal tubular dysfunction with renal tubular acidosis, nephrogenic diabetes insipidus, glucosuria, aminoaciduria, and phosphaturiaMajor feature	Ichthyosis 50	*VPS33B*, Vacuolar protein sorting 33, yeast, homolog of, b	>80 cases reported	Birth	XLR or AR

Cockayne syndrome 216400	Proteinuria, renal failure 10	Photosensitive dermatitis (overlap with xeroderma pigmentosum); xerosis cutis; anhidrosis (occasional) 75	*ERCC6* and *ERCC8*	2.7:1,000,000 births	Infancy	AR

Ehlers–Danlos syndromes (EDS) 130000	Hypoplastic kidney, sporadic	Lax, smooth, hyperextensible skin, atrophic scars, bruises 100	*COL5A1* and *COL5A2* for classic EDS	1:5,000 for all types, classical type: 1:20,000–40,000	Childhood	AD

Cranio-ectodermal dysplasia (Sensenbrenner syndrome) 218330	Interstitial fibrosis of the kidneys: thickening of the tubular basement and tubular atrophy Common. renal failure is most common cause of death	Short nails, lax skin, fine sparse hair Common	*IFT122, WDR35, WDR19, IFT43;* their protein products constitute the intraflagellar transport complex A in cilia	>40 cases reported	Birth	AR

Ectrodactyly ectodermal dysplasia-clefting syndrome 129900	Renal agenesis and dysplasia, hydronephrosis, defects in urinary tract collection system 52	Fair pigmentation, thin skin, mild hyperkeratosis. Sparse, wiry haircommon	*TP63* (homolog of tumor-suppressor gene p53), tp63	>200 patients described	Birth	AD

GAPO (growth deficiency, alopecia, pseudoanodontia, optic atrophy) 230740	Polycystic kidney Occasional	Mild skin laxity. Early alopecia Common	*ANTXR1, ANTXR1* appears necessary for actin assembly and thus cell adhesion	More than 30 patients reported	Infancy (6 months onward)	AR

**Table 2:** Syndromes with cutaneous and reno-urinary involvement.

Disorder MIM	Kidney involvement and its frequency (% of cases)	Skin involvement and its frequency (% of cases)	Affected gene (locus) and protein	Incidence	Onset of clinical manifestations	Inheritance
Adams–Oliver syndrome 1 100300	Duplicated collecting system Occasional	Aplasia cutis congenita over posterior parietal region (common), on trunk and limbs (occasional); Cutis marmorata telangiectasia congenita; thin, hypopigmented skin (occasional) 20	Gain-of-function mutations in *ARHGAP31* (identified in some affected individuals); Cdc42/Rac1 GTPase regulator	Approximately 1 in 225,000 individuals, >100 cases reported	Intrauterine/birth	AD, in few cases AR inheritance suggested

Apert syndrome 101200	Polycystic kidneys, hydronephrosis 10	Hyperhidrosis and pronounced acne (including forearms) at adolescence Common	*FGFR2;* Fibroblast growth factor receptor 2	1:80,000	Infancy	AD

Beckwith–Wiedemann syndrome 130650	Large kidneys showing renal medullary dysplasia, renal cysts, anomalies in the urinary tract collection system and resulting hydronephrosis, nephrolithiasis. Natural history: Development of neuroblastoma and Wilms tumors. Tumor formation in approximately 7.5	Facial nevus flammeus, hemihypertrophy, unusual fissures and indentations in the external ear Common	Distal arm of 11p Imprinting disorder	1:13,700	Infancy/childhood	AD

Coffin–Siris syndrome 135900	Hydronephrosis, microureters with stenoses, ectopic kidneys Occasional	Hemangiomas, hypertrichosis, hirsutism Occasional	*ARIDIB (6q25) and SMARCB1;* encode subunits of switch/sucrose non-fermenting complex, an epigenetic modifier	Approximately 140 cases reported	Birth/infancy	Probably AD

DiGeorge syndrome 188400	Congenital abnormalities of the kidney and urinary tract: single kidney, multicystic, dysplastic kidney/small kidneys, horseshoe kidney, duplicated collecting system 30	Severe acne 23%; seborrhea 35%	1.5- to 3.0-Mb hemizygous deletion of chromosome 22q11.2 including haploinsufficiency in the transcription factor gene TBX1, and CRKL which is a dosage-sensitive regulator of genitourinary development (1)	1 in 4,000	Birth/infancy	AD or sporadic resulting from *de novo* deletion

Early urethral obstruction sequence (Prune belly syndrome) 100100	Urethral obstruction (mostly due to urethral valves) leads to hydronephrosis and limits renal development 100	Excess and lax abdominal skin (“prune belly”) if bladder ruptures during fetogenesis 100	Several genes identified: *ACTA2, CHRM3, HPSE2*	1 in 29,231	Prenatal	AR

Fabry disease 301500	Chronic kidney disease, glomerular sclerosis, vacuolization of glomerular and tubular epithelial cells, renal failure	Angiokeratomas, hypo- or anhidrosis	*GLA;* alpha-galactosidase A	1:1,500–1:3,100	Variable; males develop symptoms in childhood, females >50 years	XLR

Fanconi pancytopenia syndrome 227645 227646 227650 300514 600901 603467 609053 609054 610832 613390 613951 614082 614083 615272 616435 617243 617244 617247	Renal anomalies (hypoplasia or malformation) 34	Brownish pigmentation 64	15 genes identified	1:160,000	Childhood	AR

Hajdu–Cheney syndrome 102500	Renal defects, especially cystic kidneys Occasional	Hirsutism Occasional	*NOTCH2*	NA	Childhood	AD

Nail-patella syndrome (hereditary osteo-onycho dyplasia) 161200	Glomerulonephritis, nephrotic syndrome, renal insufficiency 25	Triangular lunula, hypoplastic nails, webbing, absence of distal dorsal phalangeal skin creases Common	*LMX1B;* LIM homeobox transcription factor 1 B	1:50,000	Childhood	AD

Oral–facial–digital syndrome 311200	Polycystic kidney disease at adult age; glomerular cysts 50	Seborrheic skin, milia, alopecia Occasional	*OFD1*, involved in ciliary function	>160 cases reported	Birth	XLD

Pallister–Hall syndrome 146510	Renal ectopia or dysplasia Common	Midline facial hemangioma Common	*GLI3*	NA	Intrauterine/birth	AD

Roberts syndrome 268300	Polycystic or horseshoe kidney Occasional	Midfacial capillary hemangioma 78	*ESCO2* Essential in chromosomal alignment and adhesion in mitosis	Approximately 50 cases reported	Prenatal/at birth	AR

Robinow syndrome 268310	Renal anomalies 29	Nevus flammeus 23	*ROR2* and *WNT5A*	Sporadic cases	Prenatal	AR (*ROR2*) and AD (*WNT5A*)

Rubinstein–Taybi syndrome 180849	Renal anomalies 52	Hirsutism, capillary hemangioma, development of keloids75, 25, and 22%, respectively	*CBP* (CREB-binding protein) and *EP300*	1:100,000–1:125,000	Infancy	AD

Russell–Silver syndrome 180860	Renal anomalies, occasional	Café-au-lait spots common	*ICR1* regulator of the expression of IGF-2, and others	1:30,000–1:100,000	Infancy	Sporadic, genetically heterogeneous

Trichorhino Phalangeal syndrome (Langer–Giedion syndrome) 150230	Urethral reflux Occasional	Looseness or redundancy of skin in childhood, macularpapular nevi common	Deletion in 8q24.11–q24.13 (involving *TRPS1* and *EXT1*)	NA	Childhood	AD

### Epidermolysis Bullosa

Epidermolysis bullosa encompasses disorders defined by cutaneous and mucosal fragility. Classification into four major EB types, EB simplex, junctional EB, dystrophic EB, and the Kindler syndrome, is based on the ultrastructural level of skin cleavage (2, 3). Renal and urinary tract anomalies may occur in all EB types, in particular in junctional and dystrophic EB. In patients with severe dystrophic EB due to absence of collagen VII various renal pathologies may occur and lead to chronic renal failure. Hydronephrosis, poststreptococcal glomerulonephritis, IgA mesangial disease, or renal amyloidosis has been reported in dystrophic EB case series (4, 5). The mechanisms may include repetitive vesiculation within the lining epithelia of the urinary tract, and chronic systemic inflammation (6). Only EB types for which reno-urinary involvement is a primary feature will be described here.

#### Interstitial Lung Disease, Nephrotic Syndrome, and EB (ILNEB; MIM 614748)

##### Clinical Features

ILNEB is a rare autosomal recessive multiorgan disorder affecting the skin, kidneys and lungs. So far, 11 cases have been identified [reviewed in Ref. (5), and own unreported data], but the disease may be under recognized.

The clinical manifestations of ILNEB encompass the triad of early onset interstitial lung disease with respiratory distress, variable renal anomalies, and skin fragility. Since integrin α3 is widely expressed, clinical manifestations may occur in other organs, but are not characterized yet, because of the small number of cases and the early lethality. Skin involvement may include blistering, erosions or nail dystrophies, or may remain clinically unrecognized. The following renal anomalies were reported: congenital nephrotic syndrome, focal–segmental glomerulosclerosis, bilateral renal cysts, and a spectrum of CAKUT, including renal hypodysplasia, unilateral kidney hypoplasia, and ectopic conjoint kidney (7–12). Recently, two siblings of 13 and 9 years with viable ILNEB phenotypes presenting with growth retardation, severe pulmonary fibrosis, skin atrophy and erythema, scarce eyelashes/eyebrows, and nail anomalies (pachyonychia) but without renal features were described (13).

##### Genetics and Molecular Pathology

This disease is caused by mutations in the gene for integrin α3 (*ITGA3*) (7). Thus far, 10 *ITGA3* mutations have been reported: 2 frameshift, 2 splicing, and 6 missense mutations (5). Loss-of-function mutations were associated with lethality before the age of 2 years. The consequences of missense mutations cannot be easily predicted. Some of them were shown to disturb the posttranslational modifications of integrin α3, which proved to be critical for the heterodimerization with integrin β1 and localization to the cell membrane (8, 9, 14).

Integrin α3 is the main integrin linking podocyte foot processes to the glomerular basement membrane [reviewed in Ref. (15, 16)]. In keratinocytes, it is located at cell–matrix adhesions, promoting epidermal adhesion primarily by maintaining the integrity of the basement membrane (17). The integrin α3 subunit is a widely expressed type I transmembrane protein consisting of a large extracellular region, a single transmembrane domain, and a short cytoplasmic tail (18). It forms obligate heterodimers with β1 integrin serving as a receptor for laminins, the major components of epithelial basement membranes (19). Integrin α3 is reduced or lost in several acquired conditions with glomerular disease, in which it is associated with reduction in podocyte adhesion to the glomerular basement membrane. For example, in podocytes of early-stage diabetic nephropathy integrin α3 expression was upregulated (20), while expression was suppressed with progression of the disease (21). In patients with primary focal segmental glomerulosclerosis, podocyte depletion was accompanied by reduced podocyte expression of α3β1 integrins (22). Moreover, integrin α3 is involved in podocyte foot process effacement during nephrotic syndrome (23).

#### Nephropathy with Pretibial EB and Deafness (MIM 609057)

##### Clinical Features

Two siblings with congenital nephrotic syndrome and pretibial EB were first described in 1988 (24). The disease-causing mutation in the gene for the tetraspanin CD151 was identified in 2004 (25), and very recently an additional case was reported (26). The first two cases had proteinuria in the nephrotic range and end-stage renal failure requiring hemodialysis or peritoneal dialysis from the age of 14 or 16 years on, respectively (24). The third case was a 33-year-old male with nephropathy manifesting with proteinuria below the nephrotic range, multiple episodes of pyelonephritis, and urinary incontinence, manifesting as a combination of overflow incontinence and intermittent urge incontinence (26). Additional manifestations included pretibial or extensive skin blistering, poikiloderma, nail dystrophy, hair loss, dystrophic teeth, involvement of the ocular, oral, gastrointestinal, and urogenital mucosal membranes (25, 26).

##### Genetics and Molecular Pathology

A homozygous single-nucleotide duplication in the *CD151* gene leading to frameshift and a premature stop codon was identified in the first two cases (25). Flow cytometry analysis demonstrated absence of reactivity for CD151, suggesting that the predicted truncated polypeptide was not functional. In the third case, a homozygous *CD151* splice site mutation, affecting a canonical donor splice site junction was found (26). Immunofluorescence staining and western blot analysis confirmed that the splice site mutation led to absence of CD151 in the cells of the patient (26).

CD151 (syn. Raph blood group, TSPAN24) is a member of the tetraspanin family of cell surface proteins and acts as a stabilizer of integrins (27). CD151 forms complexes with integrin α3β1 in cell culture and *in vivo* (28, 29). These complexes are assembled early during the integrin biosynthesis and precede the interaction of CD151 with other tetraspanins (30). CD151 also regulates glycosylation of α3β1 (31). CD151 is widely expressed in epithelia, endothelia, muscle cells, renal glomerular podocytes, Schwann and dendritic cells, in platelets and megakaryocytes. CD151 is involved in the formation and/or maintenance of the glomerular basement membrane (32).

#### Junctional EB with Pyloric Atresia (MIM 226730)

##### Clinical Features

**Figure 1:**
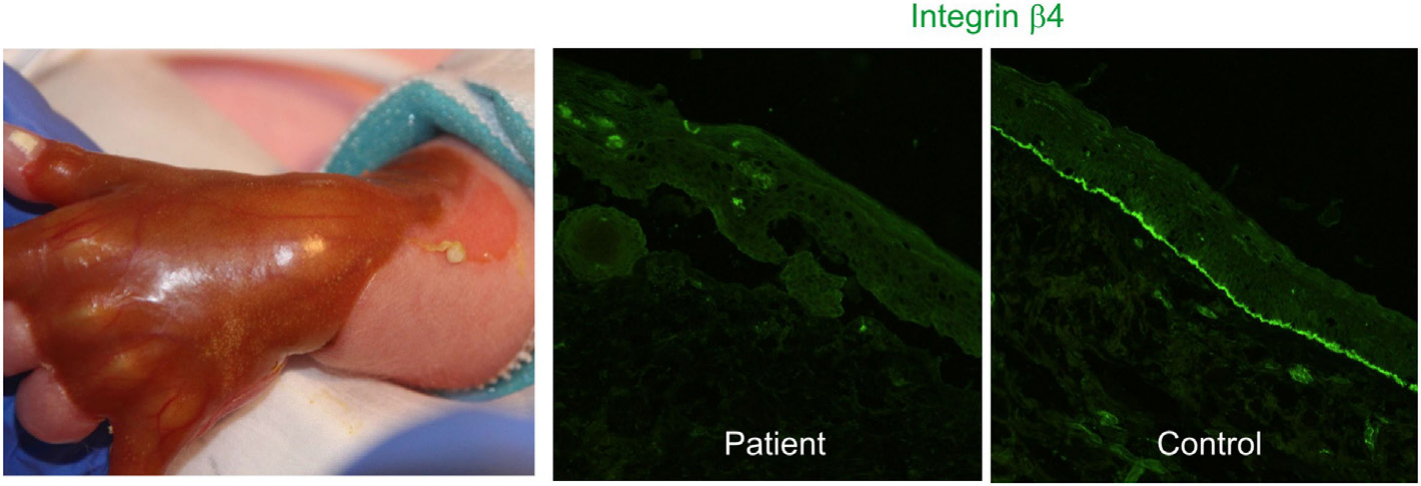
Congenital absence of the skin, also known as aplasia cutis congenita, in a newborn with hydronephrosis and pyloric atresia due to integrin α6β4 deficiency (right panel) [clinical pictures, courtesy of Dr. P. Häusermann (Department of Dermatology Basel)].

Junctional EB with pyloric atresia manifests with aplasia cutis congenita (Figure 1), generalized skin blistering, and pyloric atresia. Several acquired complications of the reno-urinary system are reported, including pyelonephritis, hydronephrosis, urinary retention, development of bladder hypertrophy, and urethral meatal stenosis (4, 33, 34).

##### Genetics and Molecular Pathology

The disease is caused by mutations in the genes coding for the α6 or β4 integrin subunits, most mutations residing in *ITGB4*. Absence of α6β4 integrin is associated with a high rate of lethality in the first months of life, while missense and splicing mutations lead to moderate disease severity and reno-urinary manifestations.

Integrin α6β4 is a heterodimer composed of two type I transmembrane subunits localized in hemidesmosomes’, which anchor keratin intermediate filaments to the cell membrane and extracellular matrix. The intracellular region of α6β4 consists of the short tail of α6 and a large β4 cytoplasmic domain, which interacts with plectin and collagen XVII in keratinocytes. The ligands of α6 integrin are CD151, collagen XVII and laminin 332. Integrin α6β4 has a major adhesive function and promotes polarization of the cells (35). α6β4 is expressed in the epithelial cells within the medulla of the kidney. In a mouse model, α6β4 was not required for morphogenesis of the urinary tract, but for maintaining the integrity of the kidney collecting system. Collecting duct anomalies appeared as the animals aged. α6-null collecting duct cells were not able to withstand mechanical stress and detached from the basement membrane (36, 37).

#### Junctional EB Generalized Severe (Formerly: Herlitz EB; MIM 226700)

##### Clinical Features

Junctional EB generalized severe is caused by complete lack of laminin 332, the major laminin expressed in the cutaneous basement membrane. Laminin 332 is a heterotrimeric glycoprotein consisting of three polypeptide chains: laminin α3, β3, and γ2, encoded by *LAMA3, LAMB3*, and *LAMC2*, respectively. The clinical picture is dominated by mucocutaneous blistering from birth onward. Extensive generalized blistering leads to loss of fluids and protein and failure to thrive. The most common complications are anemia, dyspnea, infections, and sepsis. Affected children show multiorgan involvement and commonly die before the age of 2 years (38). In an infant with *LAMB3* mutations, nephrotic syndrome with albuminuria due to failure of the glomerular filtration barrier, and high urinary *N*-acetylglucosaminidase levels, also indicating renal tubular involvement were reported (39).

##### Genetics and Molecular Pathology

Laminin 332 is the major laminin expressed by keratinocytes, but is also present in multiple epithelial basement membranes, including those of kidney. Like all laminins, it is a glycoprotein composed of three chains (α3, β3, and γ2) bound through disulfide bonds (5). In junctional EB generalized severe, mutations are found in one of the three genes encoding the laminin 332 chains. In the majority of cases, mutations are located in *LAMB3* and lead to premature termination codons, mRNA decay, and absence of laminin 332.

### RASopathies

RASopathies represent an expanding common group of neurodevelopmental disorders caused by germline mutations in genes encoding components of the Ras/MAPK pathway (40). Collectively, they affect >1 in 1,000 individuals (41). The Ras/MAPK pathway is a conserved omnipresent intracellular signaling pathway that is critical in regulating cell cycle, differentiation, growth, apoptosis, and senescence (40). The group of RASopathies includes neurofibromatosis type 1 (NF1), Noonan syndrome (NS), NS with multiple lentigines, Legius syndrome, Costello syndrome, cardio-facio-cutaneous syndrome, capillary malformation-arteriovenous malformation, and autosomal dominant intellectual disability type 5. Because of the common molecular mechanisms, phenotypic features of these syndromes are overlapping.

#### NF1 (von Recklinghausen Disease, MIM 162200)

##### Clinical Features

With an incidence of 1:2,500–3,000 (42), NF1 is one of the most common disorders of this group. NF1 follows autosomal dominant inheritance, about half of all cases occur due to spontaneous mutations. Diagnosis of NF1 is established following a set of clinical diagnostic criteria established in 1988 [Table 3, diagnosis of NF1 is probable when more than two criteria are present (43)]. Most cases are diagnosed in childhood, but when the complete set of criteria is not yet evident, follow-up and re-evaluation are necessary. Cutaneous features include café-au-lait macules, cutaneous and internal neurofibromas, or plexiform neurofibromas and axillary freckling. Renal involvement occurs sporadically, manifestations include hypertension due to renal artery stenosis, renal neurofibromas, and renal metastases of malignant schwannomas. The cooccurrence of NF1 and Wilms’ tumor has been reported in some cohorts (44, 45).

**Table 3:** Diagnostic criteria for neurofibromatosis 1 (NF1) (43).

6 or more café-au-lait macules (>0.5 cm in children or >1.5 cm in adults) 2 or more cutaneous/subcutaneous neurofibromas or one plexiform neurofibroma Axillary or groin freckling Optic pathway glioma 2 or more Lisch nodules (iris hamartomas seen on slit lamp examination) Bony dysplasia (sphenoid wing dysplasia, bowing of long bone ± pseudarthrosis) First degree relative with NF1

Individuals with NF1 have a high risk of developing malignancies, especially malignant peripheral nerve sheath tumors (46). Life expectancy has been found to be approximately 8 years lower than in the normal population (47).

The cutaneous features progress with age. Neurofibromas as the main cutaneous finding in NF1 can be itchy, lead to disfigurement, and cause psychological strain. They can be treated with excisions or laser ablation (Er:YAG or CO_2_ laser) (48, 49), both with risk for hypertrophic scarring and recurrence (42).

In uncomplicated cases, clinical evaluation in childhood should be performed annually and include auxologic measurements, cardiovascular assessment, skin examination, and developmental progress (42). In childhood, visual assessment should be performed every 6–12 months for early detection of optical pathway glioma until the age of 7 years (42).

##### Genetics and Molecular Pathology

Monoallelic loss-of-function variants in *NF1* coding for neurofibromin 1 are disease-causing in NF1. Neurofibromin is a multifunctional tumor suppressive protein which functions as a GTPase-activating protein. Neurofibromin inhibits cell proliferation and growth by blocking RAS-mediated signal transduction and modulates cell motility and adhesion (50).

The mutational spectrum is highly heterogeneous including nonsense and missense mutations, splice site mutations (about 30% of cases), small insertion–deletions, whole-gene deletions (4–5%), and structural rearrangements (51). Penetrance is complete after childhood, but NF1 is characterized by extreme clinical variability which is poorly understood, as are genotype–phenotype correlations. Intra- and interfamilial evaluation of the NF1 phenotype suggests that genetic modifiers which are not linked to the *NF1* locus contribute to the variable expressivity of the disease (52, 53). Differently skewed expression of the *NF1* alleles as well as somatic “second hit” variants or loss of heterozygosity may account in part for the phenotypic variability (54, 55). In addition to NF1, atypical manifestations, such as familial spinal neurofibromatosis, multiple spinal ganglioneuromas, optic gliomas, or Lentigines, Electrocardiographic abnormalities, Ocular hypertelorism, Pulmonary stenosis, Abnormalities of genitalia, Retardation of growth and Deafness (LEOPARD) syndrome, have been associated with *NF1* mutations. Finally, incidental occurrence of *NF1* mutations together with mutations in other genes may account for atypical phenotypic associations.

#### NS with Multiple Lentigines (syn. LEOPARD Syndrome, Multiple Lentigines Syndrome, Lentiginosis profusa and Progressive Cardiomyopathic Lentiginosis; MIM 151100)

##### Clinical Features

Noonan syndrome with multiple lentigines is a rare RASopathy that manifests in childhood. The incidence is unknown; so far, more than 200 cases were published. The characteristic cutaneous appearance is described well by the acronym LEOPARD: the skin appears spotted due to thousands of dark brown lentigines of 1–5 mm size which are distributed on the entire body (including sun-protected areas), cooccurring with café-au-lait macules (hence sometimes confused with NF1), hypomelanotic macules, and sometimes axillary freckling. Apart from LEOPARD are defining features (56). CAKUT, including horseshoe kidneys, occur in 11% of affected individuals (57). NS with multiple lentigines is sometimes difficult to distinguish from NF1 and the allelic NS (58), especially in early childhood when pigmentation anomalies are not yet pronounced (59). The prognosis is generally good, but can be limited by hypertrophic cardiomyopathy, arrhythmias, and sudden cardiac death. Annual cardiologic check-up should be performed life-long, and hearing assessment should be undertaken until adulthood. If auxologic follow-up indicates small statue, growth hormone therapy should be considered (56). Intense pulsed light has been used for cosmetic treatment of lentigines (60).

##### Genetics and Molecular Pathology

Noonan syndrome with multiple lentigines is allelic with NS and with the cardio-facio-cutaneous syndrome. The genetic basis of NS with multiple lentigines is heterogeneous including heterozygous pathogenic variants in one of four genes *PTPN11* (90% of cases), *RAF1* (less than 5% of cases), *BRAF* and *MAP2K1* (both in single cases) (61). One or more additional, as-yet undefined genes are probably associated with about 5% of cases in whom no pathogenic variant has been identified (61). Genotype–phenotype correlations are not well established (62).

All involved genes code for components of the Ras/MAPK pathway:

- *PTPN11* encodes the protein tyrosine phosphatase non-receptor type 11 that in its active form increases downstream Ras activity
- *RAF1* encodes a serine–threonine kinase that activates MEK1 and MEK2
- *BRAF* encodes the B-Raf proto-oncogene serine/threonine kinase that activates MEK1 and/or MEK2 by phosphorylation
- *MAP2K1* encodes the mitogen-activated protein kinase kinase 1 that activates ERK1 and/or ERK2 by phosphorylation (41).

Somatic mutations in all these genes are present in various types of cancers. Indeed, individuals with NS have a threefold increased risk of malignancies, such as juvenile myelomonocytic leukemia, acute lymphoblastic leukemia, rhabdomyosarcoma, and neuroblastoma (63, 64).

### Genetic Tumor Predisposition Syndromes Affecting both Skin and Kidney

This is a large group of disorders characterized by both numerous hamartomas (benign tumors that can develop in basically all tissues) and premature development of malignant tumors during childhood. The molecular pathology of these conditions is highly heterogeneous. The most common conditions are described below or in Table 4. The tumors in these syndromes can occur in both cutaneous and extracutaneous locations, including the kidney (Table 4). Other tumor predisposition syndromes which usually manifest in adult age are only briefly mentioned.

**Table 4:** Genetic tumor predisposition syndromes with cutaneous and reno-urinary involvement.

Disorder MIM	Kidney involvement and its frequency (% of cases, if known)	Skin involvement and its frequency (% of cases, if known)	Affected gene and protein	Incidence	Onset of symptoms	Inheritance
Cowden syndrome 158350	Renal cell carcinoma	Trichilemmomas, lipomas, acral keratoses, penile hyperpigmentation Main feature	*PTEN;* Phosphatase and tensin homolog	1:200,000–250,000	Adulthood	AD

Hereditary leiomyomatosis and renal cell cancer 150800	Renal cell carcinoma 10–20	Leiomyomas 76	*FH;* Fumarate hydratase	Unknown, approximately 100 families reported	>30 years onward	AD

Birt–Hogg–Dubé syndrome (syn: fibrofolliculomas with trichodoscomas and acrochordons) 135150	Renal tumors (both benign and malign) and cysts 27	Fibrofolliculomas, trichodiscomas and epidermoid cysts Common	*FLCN;* folliculin	>400 cases reported	Adulthood (>20 years), renal cancer at around 50 years	AD

Von Hippel–Lindau syndrome 193300	Renal clear cell carcinoma, renal cysts Variable	Capillary malformations, hemangioma, café-au-lait spots Variable	*VHL;* Von Hippel-Lindau tumor suppressor	1:36,000–1:45,000	Onset in adulthood	AD

#### Tuberous Sclerosis Complex (TSC, TSC1 and TSC2, syn. Bourneville Disease; MIM 191100)

##### Clinical Features

Tuberous sclerosis complex occurs with an estimated incidence of 1:5,800–1:10,000 (65). It is mostly diagnosed in infancy when it manifests with skin findings and seizures due to cerebral hamartomas and giant cell astrocytomas. The diagnosis of TSC is made according to clinical criteria (66) (Table 5, either two major features are required or, alternatively, one major and two or more minor features). Typical cutaneous features are hypopigmented macules (best seen in Wood’s light), angiofibromas (mostly facial), periungual fibromas, and connective tissue nevi (shagreen patches). The frequency of cutaneous findings increases with age, but polygonal hypomelanotic macules, known as “ash-leaf spots,” are the earliest manifestation and are invariably present at birth. Renal involvement is also common, with angiomyolipomas and cysts as the most frequent renal manifestations found in 17% of children with TSC by age 2 years and 65% of 14 years old children with TSC (67). Renal cell carcinoma is more common in TSC than in the overall population (68).

**Table 5:** Diagnostic criteria of tuberous sclerosis complex [adapted from Ref. (66)].

Major clinical features	Minor clinical features
Hypomelanotic macules (≥3, at least 5 mm diameter) Angiofibromas (≥3) or fibrous cephalic plaque Ungual fibromas (≥2) Shagreen patch Retinal hamartomas (multiple) Cortical dysplasia (≥3, including tubers and brain white matter radial migration lines) Subependymal nodules Subependymal giant cell astrocytoma Cardiac rhabdomyoma Lymphangioleiomyomatosis Angiomyolipomas (≥2)	“Confetti” lesions of the skin (hypomelanotic macules with 1–2 mm) Dental enamel pits (≥3) Intraoral fibromas (≥2) Retinal achromic patch Renal cysts (multiple) Nonrenal hamartomas

There is large variability in the clinical course, neurological development, and life expectancy in TSC, mostly depending on the number and location of hamartomas. While cutaneous features are crucial for clinical diagnosis, central nervous system tumors are the main cause of morbidity and mortality, while renal disease is the second leading cause of early death (69).

As TSC involves multiple organ systems, interdisciplinary care is necessary. Skin examinations should be performed annually. Diagnostic work-up of the kidney should include annual assessment of renal function and blood pressure and imaging (preferably with MRI) every 1–3 years (70). Since 2005, mTOR inhibitors have been evaluated for the use in TSC. Everolimus (Votubia^®^) is approved as a system therapeutic for use in children of 3 years and older with subependymal giant cell astrocytomas and for adults with complicated renal angiomyolipomas (71). Cutaneous lesions can be treated surgically, using laser (CO_2_/Erbium:YAG/Dye laser combination, or CO_2_, or Nd:YAG, or pulsed-dye laser) (72–74) and pharmacologically using topical mTOR inhibitors (75, 76). Surgical intervention can be considered as a therapeutic option for painful hemorrhagic renal angiomyolipomas and cerebral lesions.

##### Genetics and Molecular Pathology

Tuberous sclerosis complex is caused by monoallelic mutations in *TSC1* (about 20% of cases) or *TSC2* (about 70% of cases) (69) (Leiden open variation database). Two-thirds of TSC cases result from *de novo* pathogenic variants, and in about 10% no mutation can be detected (69). Large gene rearrangements, intronic pathogenic variants, and somatic or germ line mosaicism may explain the failure to detect mutations (77, 78). Specialized methods, such as targeted-deep sequencing of introns and exons and high-resolution SNP arrays improved the mutation detection rate to 94% (79). Genotype–phenotype correlations revealed that *TSC2* mutations lead to earlier onset and more severe phenotype, as compared with *TSC1* mutations (80). The occurrence of autosomal dominant polycystic kidney disease in TSC may be due to a contiguous deletion of *TSC2* and *PKD1* (81).

*TSC1* and *TSC2* code for hamartin and tuberin which form heterodimers within the TSC protein complex. Loss-of-function mutations in either *TSC1* or *TSC2* lead to constitutive activation of the mammalian target of rapamycin complex 1 (mTORC1) that is uncoupled from inhibitory mechanisms. Thus tumor cells in TSC have increased activation of mTORC1 signaling, resulting in increased protein synthesis and cell growth, and reduced autophagy (82). In fact, somatic inactivation of normal alleles is expected to drive mTOR activation, but second hit mutations are not always observed. The pathogenesis of angiofibromas involves UV-induced mutations suggesting that sun exposure is the initiating event (83). In angiomyolipomas, about 70% of the second-hit events are loss-of-heterozygosity mutations (84). A recent study showed that in TSC, somatic mutation rates were lower than most malignant tumors, while whole or arm-level chromosome gains and losses were the most remarkable finding in over 10% of patients (79).

#### Basal Cell Nevus Syndrome (syn. Gorlin Syndrome, Gorlin–Goltz Syndrome, Nevoid Basal Cell Carcinoma Syndrome; MIM 109400)

##### Clinical Features

The basal cell nevus syndrome is a rare autosomal dominant condition, occurring with an estimated incidence of 1:30,000 (85). It formally belongs to the group of hamartoses, but is mainly ranked among the tumor predisposition syndromes. Its characteristic feature is the occurrence of multiple basal cell carcinomas from young adulthood onward. Development of basal cell carcinoma in infancy has also been described (86). Other skin manifestations include palmar and plantar punctate dyskeratotic pits and facial milia. Skeletal abnormalities (e.g., polydactily), jaw cysts, and medulloblastoma occurring in 5% of patients are early features that can hint toward the diagnosis of basal cell nevus syndrome. Renal anomalies, such as renal agenesis (87) or Wilms tumors (88, 89), were reported in single cases. Diagnosis can be difficult in childhood due to few or unspecific findings. In suspected basal cell nevus syndrome, a systematic work-up including examinations by a dermatologist, a radiologist, a dentist, a gynecologist, a cardiologist, and a geneticist is recommended (90). After the occurrence of the first basal cell carcinoma, dermatologic examinations should be performed every 6–12 months. A baseline cerebral MRI with yearly controls until the age of 8 years is recommended. Echocardiography should be performed at baseline to rule out cardiac fibromas. X-ray of the jaw should be repeated yearly until a first jaw cyst is detected, after that every 6 months or according to symptoms. For scoliosis detection, an X-ray at the age of 1 year or at time of diagnosis is recommended. If normal, it is only repeated in case of symptoms. If scoliosis is present, regular follow-ups are appointed. Other baseline evaluations include pelvic ultrasound and ophthalmologic assessments. Psychological evaluation and support is advisable (90).

For many years, excision of basal carcinoma was the main treatment option for this condition. Understanding of the molecular pathology has recently led to development and approval of vismodegib (Erivedge^®^) as an effective therapy. Vismodegib targets the sonic Hh pathway and leads to regression of existing and inhibits the development of new tumors (91). Radiation should be avoided as it will trigger the eruption of multiple new tumors (92).

##### Genetics and Molecular Pathology

The genetic basis of the basal cell nevus syndrome is heterogeneous. The main cause is represented by monoallelic germline pathogenic variants in *PTCH1* responsible for approximately 85% of the cases. *SUFU* pathogenic variants reside in about 5% of the cases (93). Rare causes are *PTCH2* and *SMO* mutations: a missense mutation in *PTCH2* was disclosed in a Chinese family (94), and a *SMO* mutation in a single case with a segmental basal cell nevus syndrome (95). In about 15–27% of cases, the genetic basis remains unclear (93). Low level of postzygotic mosaicism may explain some of the genetically unsolved cases (96). *PTCH1* pathogenic missense variants have also been associated with holoprosencephaly (97).

All these genes encode key players in the Hh signaling pathway, which is essential for development of vertebrates and drives proliferation, migration, and differentiation of progenitor cells (98):

- *PTCH1* encodes the patched homolog 1, the receptor for sonic Hh
- *SUFU* encodes the suppressor of fused homolog, a negative regulator of the Hh signaling pathway
- *PTCH2* encodes patched 2
- *SMO* encodes smoothened frizzled class receptor, a G protein-coupled receptor that interacts with the patched protein.

Activation of the Hh pathway is initiated by the Hh ligand binding and inhibition of the transmembrane receptor patched 1, allowing the signal transducer smoothened to activate Gli transcription factors and amplify the expression of Hh target genes (98). Somatic mutations that activate the Hh signaling pathway drive growth of various cancers including basal cell carcinomas, medulloblastomas, pancreatic, prostate, and small cell lung cancer, that account for up to 25% of all human cancer deaths (99).

#### Birt–Hogg–Dubé Syndrome

##### Clinical Features

The Birt–Hogg–Dubé syndrome is an autosomal dominant disorder which manifests with cutaneous lesions, pulmonary cysts and/or history of pneumothorax, and various types of renal tumors (100). Skin involvement occurs during the second, third, or fourth decade of life and progresses with age. It includes various benign tumors such as fibrofolliculomas, trichodiscomas/angiofibromas, perifollicular fibromas, and acrochordons. Fibrofolliculomas are the most common phenotypic features of the Birt–Hogg–Dubé syndrome, occurring in more than 85% of the patients over the age of 25 years (101). They appear as multiple, small, skin-colored papules disseminated on the face, neck, and upper trunk. Treatment by laser ablation results in temporary improvement, but relapse usually occurs.

Individuals with Birt–Hogg–Dubé syndrome have a sevenfold increased risk to develop renal tumors, that are typically bilateral and multifocal (102, 103). They are usually diagnosed in adults (median of diagnosis is 48 years, but have been described as early as 20 years of age) and have a slow progression (103). The histologic types of renal tumors found in individuals with Birt–Hogg–Dubé syndrome are: by far predominant are chromophobe renal cell carcinomas, followed by hybrid oncocytic tumors and oncocytomas, while clear cell renal cell carcinomas are uncommon. Yearly screening by renal MRI is indicated in individuals with Birt–Hogg–Dubé syndrome age 18 years or older. In some families, renal tumors and/or spontaneous pneumothorax occur without cutaneous manifestations.

##### Genetics and Molecular Pathology

The Birt–Hogg–Dubé syndrome is caused by monoallelic pathogenic variants in *FLCN*, encoding folliculin. Mutation analysis detects disease-causing variants in 88% of the affected families; the deletion or duplication of a cytosine at position c.1285 is a mutational hot spot. Partial- or whole-gene deletions account for 3–5% of the cases and must be identified with specific methods. About 7–9% of the cases remain genetically unsolved. The protein folliculin forms a complex with folliculin-interacting protein 1 or 2 and binds to the 5′ AMP-activated protein kinase suppressing tumorigenesis (104). Moreover, it plays a role in mTOR activation (105–107).

#### Hereditary Leiomyomatosis and Renal Cell Cancer (HLRCC)

##### Clinical Features

Hereditary leiomyomatosis and renal cell cancer is characterized by the occurrence of cutaneous and uterine leiomyomata, and/or a single, unilateral, and aggressive renal tumor (108). Cutaneous leiomyomata may be multiple or single, appear in adults (mean age of 25 years), and increase in size and number with age. They manifest as skin-colored papules or nodules, disseminated on the trunk, extremities, and face. The treatment consists of surgical or laser excision, or cryoablation. Renal tumors occur in about 10–16% of individuals with HLRCC at a median age of 44 years and cause hematuria and lower back pain. Histologically they are classically classified as type 2 papillary (108).

##### Genetics and Molecular Pathology

Hereditary leiomyomatosis and renal cell cancer is caused by monoallelic *FH* mutations that lead to reduced activity of the enzyme fumarate hydratase (109). In tumor tissue, somatic variants and loss of heterozygosity are found. No genotype–phenotype correlations are known, and there is significant intrafamilial variability. Biallelic mutations resulting in fumarase deficiency cause an inborn error of metabolism characterized by rapidly progressive neurologic impairment including hypotonia, seizures, and cerebral atrophy (110).

## Disorders Due to Postzygotic Mosaicism

The disorders in this group are caused by mutations that are mostly lethal if occurring as a germline mutation affecting all cells. However, if these mutations occur postzygotic in early embryogenesis, disorders with unilateral or segmental manifestations result, as proposed by Happle (111, 112). His hypothesis is supported by the elucidation of the molecular basis of several segmental disorders since the implementation of next-generation sequencing technologies.

### Linear Sebaceous Nevus Sequence [Schimmelpenning–Feuerstein–Mims Syndrome, Nevus Sebaceous of Jadassohn; MIM 163200]

#### Clinical Features

This syndrome belongs to the group of epidermal nevus syndromes. More than 100 sporadic cases have been described, the incidence is not known. While solitary sebaceous nevi are a reasonably common finding in infants, the sebaceous nevi in this syndrome are associated with seizures, mental retardation, skeletal and ophthalmic anomalies, and asymmetric growth. At birth, one (or multiple) sebaceous nevi is/are found mostly in the mid-face. Involvement of the head/neck area is possible, as are locations on trunk or extremities. The sebaceous nevus shows a linear configuration along the lines of Blaschko. While it is mostly flat and wax-like in infancy, verrucous changes, hyperpigmentation, hyperkeratosis, and hypertrophy are seen toward puberty. In adulthood, development of (mostly benign) tumors within the sebaceous nevus is noted. Skeletal, ophthalmic, and renal involvements occur occasionally, the latter comprising CAKUT, such as double urinary collecting system and horseshoe kidneys, and renal hamartoma and nephroblastoma.

Surgical treatment of sebaceous nevi can be offered for cosmetic or psychological reasons. Excision is generally not indicated because of cancer prophylaxis, as the risk for malignant tumors is very low (113). Therapeutic options include excision and laser ablation (114). Regarding the main complications, seizures, neurological retardation, and rickets, interdisciplinary care for children with linear sebaceous nevus sequence should be offered.

#### Genetics and Molecular Pathology

This syndrome can be considered a mosaic RASopathy because it is caused by postzygotic pathogenic variants in *HRAS* (HRas Proto-Oncogene, GTPase), *KRAS* (KRAS Proto-Oncogene, GTPase), or the *NRAS* (NRas Proto-Oncogene, GTPase) genes (115, 116). The recurrent activating mutations induce constitutive activation of the MAPK and PI3K/AKT signaling pathways. Somatic mutations in the *HRAS* and/or *KRAS* genes were also found in isolated sebaceous nevi. These mutations are only present in keratinocytes which give rise to the cutaneous lesions.

The proteins encoded by the Ras oncogene family have intrinsic GTPase activity and function in signal transduction pathways important for cell growth, proliferation, and survival. Defects in these genes are present in various cancers.

### Neurocutaneous Melanocytosis (syn. Neurocutaneous Melanosis Sequence, Neuromelanosis; MIM 249400)

#### Clinical Features

The landmark lesion of this syndrome, a giant pigmented nevus, is seen at birth. It is usually located in the posterior head or trunk (117). Sometimes, three or more large congenital nevi are found rather than a single giant nevus. Numerous disseminated (“satellite”) nevi can be present at birth and more will develop in the course of disease. The presence and proliferation of melanin-producing cells within cranium and spine leads to increased intracranial pressure, seizures, mental deterioration, and death in early childhood (117). Leptomeningeal and intracranial melanoma occur in a significant portion of patients. Occasional abnormalities found in neurocutaneous melanocytosis are cerebral malformations such as syringomyelia and Dandy–Walker malformation, CAKUT, and unilateral renal cysts. Other tumors occurring in the syndrome include rhabdomyosarcoma, liposarcoma, and malignant peripheral nerve sheath tumors.

There is a risk of development of cutaneous malignant melanoma within the congenital nevi. They develop in the depth of the lesion and can be felt earlier than they can be seen. This has been suggested to be as high as 15% (118) in giant congenital melanocytic nevi, although others have reported incidences of 0.7% (119). The amount of patients with the full picture of neurocutaneous melanocytosis developing malignant melanoma is not known, probably as most of these patients die before developing melanomas.

They clinical course is mostly determined by neurologic symptoms. If these occur, there is no effective therapeutic approach. If the child shows normal psychomotoric development, excision for the giant melanocytic nevi is recommended. Such surgical procedures may require several steps and the use of tissue expanders. Dermabrasio is not considered as a therapeutic option any more. To date, a causal therapy is not available, but a recent *in vitro* assessment of inhibitors of the NRAS-signaling pathway (drugs also successfully used in the therapy of malignant melanoma) showed promising results (120).

#### Genetics and Molecular Pathology

Somatic oncogenic missense mutations affecting codon 61 of the *NRAS* gene were identified in affected cutaneous (melanocytes) and nervous tissues from patients with congenital melanocytic nevus syndrome and/or neurocutaneous melanosis (118).

### CHILD Syndrome (Congenital Hemidysplasia with Ichthyosiform Erythroderma and Limb Defects; MIM 308050)

#### Clinical Features

**Figure 2:**
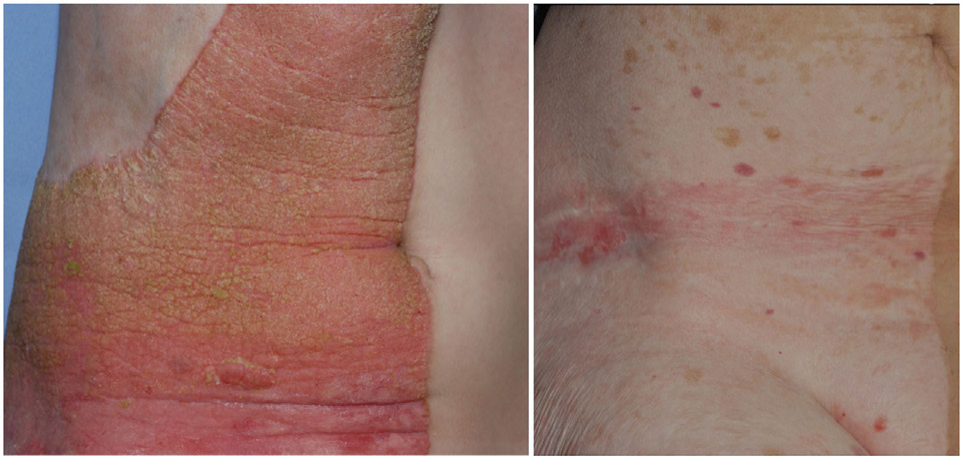
Unilateral epidermal nevus in a patient with CHILD syndrome, before (left panel) and after 5 years topical application of a simvastatin/cholesterol cream (right panel).

This epidermal nevus syndrome was coined with the acronyme “CHILD” by Happle and colleagues in 1980 (121) to sum up the main findings in children with this condition: a characteristic, mostly unilateral epidermal nevus in combination with ipsilateral congenital hemidysplasia of bones (affecting any part of the body, mainly limbs). The epidermal nevus is usually present at birth but can also develop in the first weeks of life. Spontaneous involution is sometimes witnessed (113). The CHILD nevus is red and scaly. It can show strict lateralization (right side more frequently than left side, 3:2) and midline demarcation, but it can also follow lines of Blaschko, and both patterns may be present in an affected individual (113) (Figure 2). Next to cardiovascular anomalies, renal findings comprise CAKUT, such as renal agenesis (122) or hypoplasia (123), to unilateral hydronephrosis, but their frequency is unknown (124).

Addressing the molecular pathology of CHILD, a therapeutic approach for treating the epidermal nevus combining simvastatin and cholesterol for topical use proved to be effective (125, 126) (Figure 2).

#### Genetics, Molecular Pathology

CHILD syndrome is caused by monoallelic loss-of-function pathogenic variants in the *NSDHL* gene encoding the NAD(P)H steroid dehydrogenase-like protein, which is a C4 demethylase involved in postsqualene cholesterol biosynthesis (127). The enzyme is located within the membranes of the endoplasmic reticulum. Its deficiency leads to impaired cholesterol processing, causing abnormal sonic Hh signaling, which affects spatial patterning during embryogenesis (128). The cutaneous features may result from a dual mechanism: accumulation of cholesterol precursors and cholesterol deficiency (128).

This X-linked dominant disorder is lethal in male during gestation and thus predominantly affects females. The CK syndrome [initials of the original proband (129)] is an X-linked recessive disorder that affects males being also caused by pathogenic *NSDHL* variants (130). In CHILD syndrome, mosaicism results from inactivation of an X-chromosome in females. Inter-individual differences in the pattern of X inactivation explain the phenotypic variations.

### Focal Dermal Hypoplasia (Goltz syndrome; MIM 305600)

#### Clinical Features

Focal dermal hypoplasia is rare; more than 175 cases have been reported. The focal dermal hypoplasia is mostly encountered in females (90%), as its X-linked dominant inheritance leads to lethality in male fetuses. Affected males usually show a mosaic form of focal dermal hypoplasia. This syndrome is evident at birth, when skin and skeletal symptoms are predominant. Children with focal dermal hypoplasia show skin atrophy with Blaschko linear arrangement, appearing as depressed or slightly raised red macules. This finding explains the original name “focal dermal hypoplasia” (131). Over the course of disease, fatty tissue can herniate through gaps in the underdeveloped connective tissue forming lipomatous papules. Papillomas and angiofibroma occur on the face and in the urogenitoanal region (132). Additional findings are patchy alopecia and thin hair. Affected children show facial dysmorphies and asymmetric skeletal deformities (e.g., syndactyly, polydactyly, amelia, scoliosis). Renal anomalies occur occasionally and include horseshoes kidneys and hydronephrosis.

No specific therapy for focal dermal hypoplasia exists. Papillomas can be surgically removed, but may reoccur.

#### Genetics and Molecular Pathology

Focal dermal hypoplasia is an X-linked dominant disorder which reflects mosaicism resulting from inactivation of an X-chromosome in females. The pathogenic variants affect *PORCN* (133). *PORCN* is a gene of the porcupine family, which code for endoplasmic reticulum proteins with multiple transmembrane domains involved in the processing of Wnt (wingless and int homolog) proteins. Mutations in different players of the Wnt signaling pathway have been described before to cause CAKUT (134), explaining the pathogenesis of CAKUT in focal dermal hypoplasia. The disease is lethal in males; live-born affected males are rare and nearly always have somatic mosaicism for a *de novo* postzygotic pathogenic variant. Postzygotic mutations may also cause mild disease in females (135).

## Chromosomal Aberrations

In case of chromosomal aberrations, e.g., deletions or trisomies, a large number of genes are affected by the defect. Therefore, the resulting clinical picture is broad and includes renal and cutaneous anomalies in some syndromes (Table 6). However, these are not defining for the clinical picture.

**Table 6:** Chromosome anomalies with cutaneous and reno-urinary involvement.

Disorder MIM	Kidney involvement and its frequency (% of cases)	Skin involvement and its frequency (% of cases)	Affected gene and protein	Incidence	Onset of symptoms	Inheritance
Microdeletion 17q21 syndrome (Koolen–De Vries syndrome) 610443	Hydronephrosis, pyelectasis, renal dysplasia and duplex renal system 32	Altered pigmentation of hair and skin, hyperelastic skin, thickened skin in some areas, hyperpigmentation of nevi 55% (hair anomalies)	Microdeletion within chromosome 17 (17q21.31) involving *KANSL1, a* chromatin modifier gene	1:16,000	Birth	AD

Trisomy 18 (Edwards Syndrome)	Horseshoe kidneys, ectopic kidney, double ureter, hydronephrosis, polycystic kidneys. Wilms tumor10–50% (Wilms tumor in <10%)	Redundant skin, cutis marmorata, hirsutism (especially at forehead) >50%	Trisomy 18	1–9/1,000 000	Birth	Random, mosaicism

Deletion 2q37 syndrome 600430	Kidney and urinary tract anomalies, Wilms tumors <5%	Eczema	Subtelomeric deletion in chromosome 2	>100 patients described	Variable	AD

Deletion 18q syndrome 601808	Horseshoe kidney, ureteral reflux occasional	Skin dimples (knuckles, shoulder), eczema occasional	Deletion of long arm of chromosome 18	1:40,000	Infancy	AD

Killian/Teschler–Nicola syndrome (=Pallister–Killian syndrome) 601803	Persistence of urogenital sinus/cloaca occasional	Streaky hypo- and hyperpigmentation, abnormal sweating occasional	Tissue-limited mosaicism with partial trisomy due to isochromosome of Chromosome 12p	5.1 per million live births	Neonatal period	Somatic mosaicism

Microdeletion 3q29 syndrome 609425	Horseshoe kidneys occasional	Abnormal skin pigmentation occasional	Microdeletion 3q29	>20 cases described	Usually onset in childhood	Unclear, only isolated cases described

*AD, autosomal dominant; AR, autosomal recessive; NA, not available; XLD, X-linked dominant; XLR, X-linked recess*.

## Differential Diagnosis in Newborns

Several acquired conditions affect both skin and kidney either pre- or postnatally. For example, maternal intake of valproate leads to fetal valproate syndrome commonly showing hemangiomas, altered pigmentation, and occasional renal malformations. Intake of phenytoin during pregnancy causes fetal hydantoin syndrome, in which hirsutism and coarse hair are common and renal malformations can occur. The oligohydramnios sequence (Potter syndrome) arises from lack of amniotic fluid. This anhydramnion or oligohydramnion can either be caused by primary renal problems such as agenesis, severe polycystic kidney deformation or obstruction of the urinary tract, or by chronic leakage from the amniotic sac. Fetal development, especially of the lungs, and life expectancy are severely limited.

## Conclusion

Genetic disorders affecting the skin and the kidneys cover a broad range of phenotypes and molecular mechanisms, which have been largely uncovered in the last decades. Many of these conditions comprise involvement of multiple organs and systems. Although, in many cases, the cutaneous findings (e.g., café-au-lait spots, angiofibromas, nevi) have no significant impact on the prognosis, they represent precious signs for the clinical diagnosis and should alert pediatricians to carefully evaluate the patients.

- Because of the clinical complexity these patients require an interdisciplinary care, comprising geneticists, dermatologists, nephrologists, cardiologists, etc., in which the pediatrician has a central coordinating role.
- The rarity of these disorders renders their diagnosis sometimes difficult, underrecognized and delayed. In addition, the cutaneous lesions have an esthetic impact (e.g., angiofibromas of the face, neurofibromas, nevi) and the tumors have an unpredictable, mostly progressive course. All these factors have a high psychological impact on the patients and their families, who require psychological aid and support from patients’ organizations.
- Illumination of the molecular pathomechanisms of some rare disorders (e.g., Ras/MAPK, mTOR, cholesterol biosynthesis) opened new opportunities to repurpose known drugs and use them to slow down disease progression. Such evolving therapies, either in the clinical practice or in clinical trials, have been briefly outlined in this review.

## Author Contributions

AR wrote most of the clinical part (clinical features) and the tables. YH prepared the figures and reviewed the manuscript. CH drafted the manuscript, wrote the genetics and molecular part, and reviewed the entire manuscript.

## Conflict of Interest Statement

The authors declare that the research was conducted in the absence of any commercial or financial relationships that could be construed as a potential conflict of interest.

## Abbreviations

CAKUT, congenital abnormalities of the kidney and urinary tract; EB, epidermolysis bullosa; Hh, hedgehog; HLRCC, hereditary leiomyomatosis and renal cell cancer; LEOPARD, Lentigines, Electrocardiographic abnormalities, Ocular hypertelorism, Pulmonary stenosis, Abnormalities of genitalia, Retardation of growth and Deafness; NF1, neurofibromatosis type 1; NS, Noonan syndrome; TSC, tuberous sclerosis complex.
